# Development and validation of the patient reported outcomes questionnaire of children with asthma in China: A Caregiver's proxy-reported measure

**DOI:** 10.3389/fped.2023.1114289

**Published:** 2023-03-23

**Authors:** Tong Xu, Haiyue Zhang, Yuhai Zhang, Peng Yang, Zhe Yang, Xun Jiang, Lei Shang

**Affiliations:** ^1^ Department of Health Statistics, School of Public Health, Fourth Military Medical University, Xi’an, China; ^2^ Department of Pediatrics, Tangdu Hospital, Fourth Military Medical University, Xi’an, China

**Keywords:** children with asthma, patient-reported outcomes, questionnaire, reliability, validity, discriminative ability

## Abstract

**Background:**

Research on asthma control levels and quality of life is essential for children with asthma during their growth stage. Therefore, it is necessary to develop a questionnaire that can be used for monitoring and evaluating the disease control effectiveness and quality of life of children with asthma in China and to conduct a preliminary evaluation for its reliability, validity, and discriminative ability.

**Methods:**

The questionnaire was created through a literature review and qualitative interviews for a targeted population. Based on the previous work, 30 caregivers of children with asthma and 5 experienced pediatricians reviewed and discussed a collection of items. Then, 72 items were screened and selected to form the draft questionnaire. After three rounds of investigation (with 240, 503, and 360 participants, respectively), the final questionnaire was established according to the evaluation results. The structure of the questionnaire was explored through confirmatory factor analysis. Exploratory factor analysis and variability analysis were applied based on the first two rounds of investigation. Reliability, construct validity, and discriminative ability were evaluated based on the third round of investigation.

**Results:**

The questionnaire contains 6 dimensions and 34 items, and the total cumulative variance contribution rate was 54.96%; Cronbach's *α* coefficient was 0.91; the split-half reliability coefficient was 0.75, and the test–retest reliability coefficient was 0.74. The children's age, gender, residence, asthma attack in the last three months, caregivers' education background, and monthly income per caregiver were correlated with patient-reported outcomes of children with asthma.

**Conclusion:**

The questionnaire appeared to have good reliability, construct validity, and discriminative ability in children with asthma in China.

## 1. Introduction

Asthma is a common multifactorial chronic respiratory disease. According to the report of the Global Initiative for Asthma (GINA) committee, the proportion of asthmatics in the population of different countries ranges from 1% to 18%. At least 300 million asthmatics exist in the world, of which 30 million are in China (1, 2). Asthma is the most common childhood chronic illness worldwide. Repeated asthma attacks severely affect children's health and social or daily life, causing massive economic burden and mental pressure on families and occupying huge medical and health resources (3). In recent years, the prevalence of childhood asthma worldwide has been significantly rising, especially in developing countries. In 2006, GINA put forward the concept of asthma control (4), emphasizing that asthma treatment aims to achieve “General Control of Asthma”. “Asthma Control” is an important part of clinical diagnosis and treatment of asthma in various asthma diagnosis and treatment activities, including the World Asthma Day. However, although the effect of asthma control has been improved, the overall control rate of asthma is not promising. A study of 988 participants on the disease control of children with asthma under 16 showed that 53.4% had poor control effects, 44% achieved partial control, and only 2.5% achieved effective control (5).

The prevalence of asthma is increasing yearly with low control. Meanwhile, children with asthma have higher morbidity and mortality than adults, making it one of the most important risk factors threatening children's health (6). The etiology of asthma is complex, involving many interacting factors, such as physiological, psychological, and social factors. Children's understanding of the disease, the monitoring, and the parents' or caregivers' ability to care for them will also affect asthma control. Therefore, it is of great significance to accurately identify the treatment and control, and take reasonable measures to improve asthma control and the children's quality of life (7).

The evaluation of clinical efficacy is mainly based on subjective judgment, objective examination, and laboratory test index. However, there are only a few quantitative measurements and evaluation criteria for patients' self-feedback. Moreover, the disparity in personal knowledge reserves, clinical experience of doctors, and patients' cognition also lead to the differences in clinical efficacy evaluation and patients' satisfaction with efficacy. With the transformation of the medical model from biomedical to bio-psycho-social medical, people's understanding of health and disease measurement and treatment effect evaluation have changed a lot. Hence, apart from medical staff and patients' biological report, patients' self-reports plays a vital role in disease diagnosis and treatment nowadays, which can be called Patient Reported Outcomes (PRO) (8).

Some studies have been reported on the questionnaire or measurement tools related to the reported outcomes of children with asthma. The Pediatric Asthma Quality of Life Questionnaire (PAQLQ) (9), developed by Juniper et al., was widely used to evaluate the daily condition of children with asthma in three dimensions: symptom, activity limitation, and emotional function. Both investigators and children with asthma completed the questionnaire. The Childhood Asthma Questionnaire (CAQ) (10, 11) was designed in different dimensions and items for children with asthma in different growth stages, which were Quality of Life and Bad Mood for 4–7 year-old, Positive Quality of Life, Negative Quality of Life, Bad Mood and Severity for 8–11 year-old. For 12–16 year-old children, a Reactive dimension was added to the questionnaire. The Children's Health Survey for Asthma (CHSA) (12) measures the physical health, activity, and mental health of 5–16 year-old children with asthma. CHSA includes two versions for child and their parents. The Asthma Symptoms and Disability Questionnaire (ASDQ) (13) was used to measure the symptoms and disability of 5–14 year-old children with asthma, including daytime and nighttime symptoms. The Pediatric Quality of Life Inventory (PedsQL) (7, 14–16), a widely used questionnaire for children's quality of life, is divided into four subscales for 2–4 year-old, 5–7 year-old, 8–12 year-old and 13–18 year-old, respectively. The subscale for 2–4 year-old children will be filled by their parents, and the rest can be filled by either the child or the parents. The questionnaire includes general dimensions such as physiological, emotional, social, and role function, as well as specific modules for children with different conditions. It developed the asthma module for children with asthma, including asthma symptoms, treatment, anxiety, and communication dimensions. The Childhood Asthma Control Test(C-ACT) (17) and the Asthma Control Questionnaire(ACQ) (18) are two widely used questionnaires developed to measure asthma control, including several items. All the measurement tools mentioned above can be categorized into two groups: PAQLQ, CAQ, CHSA, and general dimension of PedsQL are used to measure children's quality of life; C-ACT, ACQ, ASDQ and asthma dimensions PedsQL are used to measure asthma control. The applicable population is different for this study, including age and lifestyle.

In China, measurement tools to evaluate the quality of life of children with asthma are under-utilized, and most of the tools are imported from overseas, such as C-ACT, ACQ, and PedsQL. It brings some challenges in practical use, such as poor applicability, low reliability, and validity. There is no measurement and evaluation tool based on the characteristics of Chinese regional culture and the consideration of diagnosis and treatment of children's asthma. Therefore, it was necessary to develop a measurement tool with good reliability and validity for the reported outcomes of children with asthma, which can provide a suitable measurement tool for the monitoring and evaluation of disease control and quality of life of children with asthma in China. It can also provide a reference for the development of intervention strategies for children with asthma. Since the targeted population of this study is preschool children with asthma with less ability to accurately express or report their real situation, the reports will be given to children's caregivers to ensure that the measurement tool is more accurate and practical used.

## 2. Materials and methods

### 2.1. Participants

**Figure 1:**
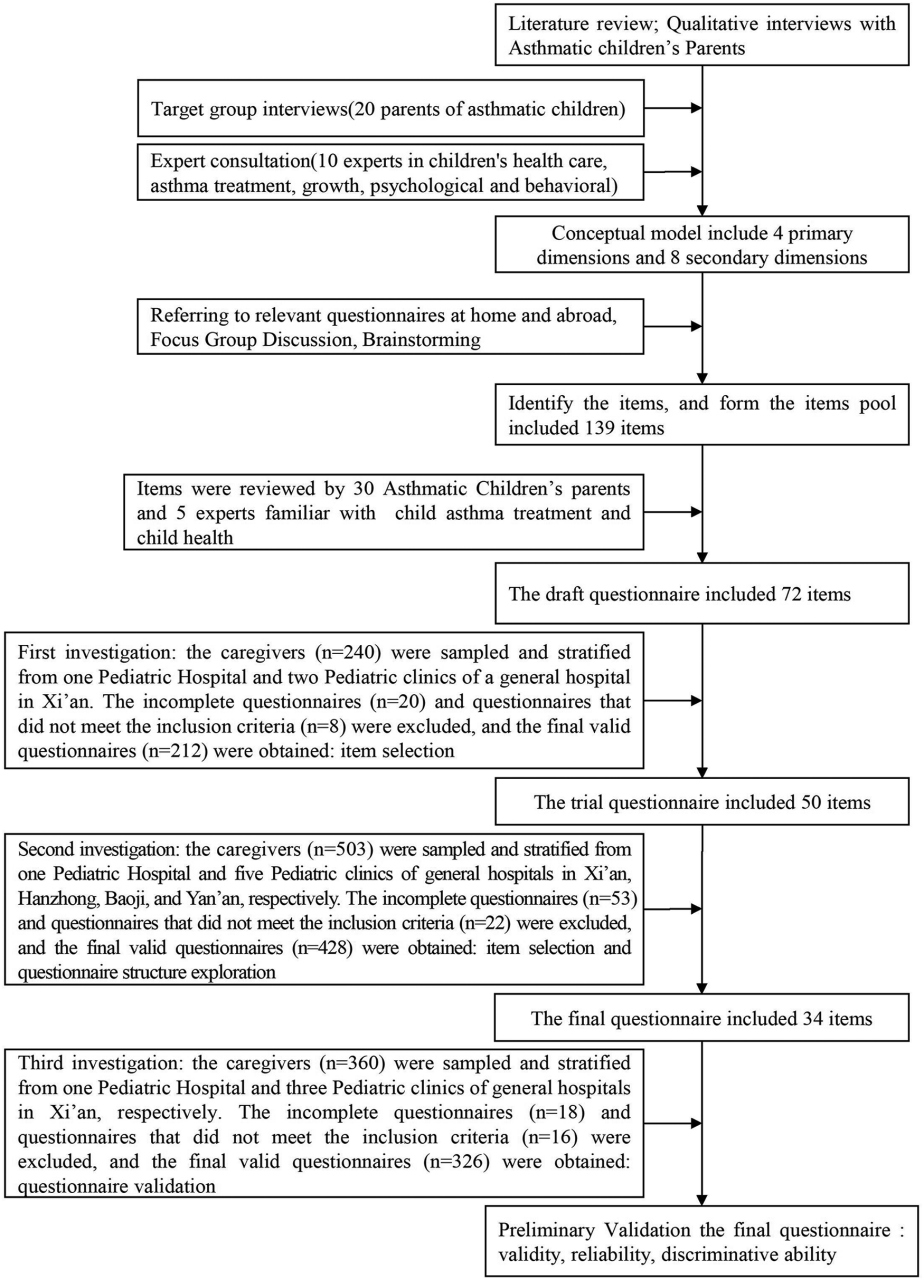
The flowchart of the research process of development and preliminary validation of patient reported outcomes questinnaire od children with asthma in China: A caregiver's proxy-reported measure.

The study was conducted from March to October 2018 in four cities in Shaanxi Province, China. To obtain representative samples of children aged 2–7 year-old with asthma, the researchers contacted children's caregivers in the pediatric outpatient department of 6 large hospitals in the 4 cities. To provide an overview of the study procedure, a flowchart of the research process is shown in Figure 1.

The caregiver was the primary person who takes care of child's daily living (e.g., diet, sleeping, activity) at home (i.e., after school and over the weekend). Caregivers would be most familiarized with their children's disease status. The following inclusion criteria were set: his or her child was 2–7 years old with exclusively asthma disease and at least a second visit to the hospital with a previous diagnosis of asthma, the caregiver agreed to participate in the survey, and the child did not have any other chronic diseases except for asthma that might influence his or her quality of life in the previous two months. Caregivers were excluded from the study if they were illiterate or reluctant to participate.

The study included different versions of the Patient Reported Outcomes questionnaire and questionnaire for general demographic information, including the child's age, gender, residence, one child's family or not, asthma flare-up, the caregivers' educational levels, and family monthly income. The selected caregivers were asked to come to a room to complete the questionnaire under the instruction of a researcher. A standardized explanation of the study's aims and procedures was provided before the formal investigation so that each caregiver could fully understand the purpose and significance of the investigation, the meaning of each item, and the instructions for completing the questionnaires.

### 2.2. Conceptual model and draft questionnaire development

The conceptual model of the questionnaire was developed after the relevant domestic and international literature was reviewed, focusing on Quality of life, patient-reported outcomes, and Clinical evaluation of asthma in children. Then, targeted group interviews (including 20 caregivers of children with asthma with good understanding and expression ability, the experienced outpatient doctor was selected as the group moderator after professional training) were used to collect patient information and reported outcomes of children with asthma. After this, expert consultation (including 10 experts in children's health care, asthma treatment, growth, and children's behavioral psychology) was used to review the rationality and completeness of the information obtained above. Then, we summarized the concept connotation and composition of the children with asthma parents' reported outcomes and formed the initial conceptual model. The conceptual model consisted of four primary dimensions (children's physiological status, psychological and behavioral status, asthma's influence and limitation on children, asthma's treatment control and influence on family) and eight secondary pre-dimensions (physical status, growth, and development, motor ability, communication and cognition, psychological behavior, asthma symptoms, asthma limitation, asthma treatment, and influence).

Based on referring to relevant questionnaires, including CHSA, ASDQ, PedsQL, C-ACT, ACQ, PAQLQ, and CAQ, we used group focus discussion methods (organized a total of 3 times in one group, including 6 research team members) to fill in all dimensions of the conceptual model to ensure that items could cover the connotation and scope of the conceptual model. Moreover, two linguists and two psychologists were invited to modify the items' contents through interviews. Finally, an item pool with 139 items was formed.

The item pool was then reviewed by 30 caregivers of children with asthma who were selected from the outpatient departments of pediatrics of the largest hospital in Xi'an City and 5 experts with expertise in child asthma treatment and child health. Each item was critically evaluated. An item would be deleted when it was ambiguous or had duplicated meaning with other items. After being reviewed by caregivers, deletions and modifications were reviewed by five experts to ensure that the items deleted were reasonable. After the review, 67 items were deleted, and 9 were modified. Ultimately, the Patient Reported Outcomes Questionnaire of Children with Asthma (ACPRO) included 72 items.

### 2.3. Scoring methods

Each item of the questionnaire measured the frequency of the performance of children with asthma during the previous month. Five option levels were determined for different items through response dimensional analysis. Namely, never, rarely, sometimes, often, and always, they were assigned a number value of 0, 1, 2, 3, or 4, respectively. If the meaning of an item was a negative description of children's health, it was reversed. The mean score was calculated as the sum of the items divided by the number of items answered in each dimension, and the mean of the scores in each dimension was the questionnaire's score. Higher scores for each dimension indicated better performance, better health status or quality of life, mild symptoms, and better disease control effects for children with asthma in this dimension.

### 2.4. Investigation methods

A series of three investigations were conducted by 2 investigators who were trained before the start of the study. First, the investigators explained the purpose and procedures for the investigation and the meaning of the questionnaires to caregivers who agreed to participate in this study. Then, the questionnaires were distributed to the caregivers. Based on their children's performance over the past month, the caregivers completed their questionnaires and returned them to the investigators.

### 2.5. Forming the trial questionnaire (first investigation)

Sample 1 consisted of 240 caregivers sampled and stratified from Xi'an Children's Hospital (*n* = 122), Pediatric clinics of the Second Affiliated Hospital of Xi'an Jiaotong University (*n* = 63) and Tangdu Hospital (*n* = 55), with the predefined investigation methods and inclusion/exclusion criteria. The caregivers independently completed the first draft of the ACPRO and returned them to the investigators. This sample was used for item analyses of the draft questionnaire and construction of the trial ACPRO.

### 2.6. Forming the final questionnaire (second investigation)

Sample 2 consisted of 503 caregivers sampled and stratified from Xi'an Children's Hospital (*n* = 178), Pediatric clinics of the Second Affiliated Hospital of Xi'an Jiaotong University (*n* = 46), Tangdu Hospital (*n* = 75), Hanzhong People's Hospital (*n* = 80), Baoji People's Hospital (*n* = 52), and Yan'an People's Hospital (*n* = 72), respectively, with the same method and inclusion/exclusion criteria used for Sample 1. The caregivers independently completed the trial questionnaire of the ACPRO and returned them to the investigators. This sample was used for item analyses of the trial questionnaire and construction of the final ACPRO.

### 2.7. Evaluating the final questionnaire (third investigation)

Sample 3 consisted of 360 caregivers sampled and stratified from Xi'an Children's Hospital (*n* = 208), Pediatric clinics of the Second Affiliated Hospital of Xi'an Jiaotong University (*n* = 53), Tangdu Hospital (*n* = 47), and Xijing Hospital (*n* = 52), respectively, with the same method and inclusion/exclusion criteria as Sample 1. The caregivers independently completed the final questionnaire of the ACPRO and returned them to the investigators. This sample was used to analyze the questionnaire's reliability, validity, and discriminative ability. A subset of 60 caregivers was randomly selected to answer the questionnaire again after 2 weeks. In this investigation, we also investigated asthma control status to monitor the disease state. The control of asthma was determined by the classification of children's asthma symptom control level in the Guideline for the diagnosis and optimal management of asthma in children (2016) (19). We added this content in the process of investigation.

### 2.8. Quality control methods

The investigators carefully double-checked all questionnaires, and all completed questions determined to be valid were entered into a database built using EpiData software. The double-entry method was used to ensure the accuracy of the data, and a logic check for errors was performed. Then, the questionnaire database was imported into SPSS 23.0 software for analysis.

### 2.9. Statistical methods

#### 2.9.1. Item analysis

First, items were discarded when more than 15% of the caregivers gave the highest or lowest score, indicating ceiling effects or floor effects (7). Second, reverse scoring items were converted using the following rules (4 = 0, 3 = 1, 1 = 3, and 0 = 4). Then, the following 5 methods were used for the item selection (20). (1) The critical ratio analysis method: by computing and sorting the total score of the questionnaire, the critical scores of the upper and lower groups were found to be 27%. The questionnaire was then divided into two groups, according to the critical scores; an independent t-test was used to distinguish the difference between the high and low groups for each item, and items were discarded if they had a *P*-value > 0.05. (2) The discrete trend method: items with a standard deviation of less than 0.85 (from the score) were discarded. (3) The correlation coefficient method: items for which the Pearson's correlation coefficient was assessed as <0.4 (by correlating each item with the total score) were discarded (21). (4) The exploratory factor analysis (EFA) method (22): items with factor loading values <0.4 were discarded. (5) Cronbach's *α* coefficient method (23): Cronbach's *α* coefficient was calculated for all items, and items that reduced the overall alpha level were discarded.

Based on these methods, the item exclusion principles were identified: an item was deleted when it was selected to be discarded by greater than or equal to 3 methods. If an item was selected by two methods, it will be deleted or merged, according to professional knowledge and expert opinions.

#### 2.9.2. Reliability analysis

Reliability analysis was evaluated by computing Cronbach's *α* coefficient, test–retest reliability coefficient, and split-half reliability coefficient. Alpha coefficients for the total questionnaire and dimensions greater than or equal to 0.70 and 0.60, respectively, were considered satisfactory (23).

#### 2.9.3. Validity analysis

The experts' consultation method was used to evaluate the content validity of the questionnaire. The content validity ratio (CVR) is calculated to determine whether the content validity meets the requirements. The calculation formula follows CVR = (ne − N/2)/(N/2). ne = The number of experts who believe that a certain item represents the corresponding test content; *N* = The total number of experts participating in the evaluation. Record the time taken to complete the questionnaire to determine whether the questionnaire is easy to understand. A two-step strategy of model building was used to assess the construct validity. Pearson correlation analysis was used to calculate the correlation coefficient between each dimension and the total score of the questionnaire. If the correlation coefficient between dimensions was smaller than that between dimensions and total score, it suggests that dimensions are independent of each other and can represent the questionnaire. To conduct confirmatory factor analysis to fit the factor model formed by exploratory factor analysis and to determine how well the factor model fit for each sample data, the investigators focused on seven fit indices, following methods described by Hu & Bentler (24, 25): the *χ*^2^/*df*, the non-normed fit index (NNFI), the comparative fit index (CFI), the goodness-of-fit index (GFI), the adjusted goodness-of-fit index (AGFI), and the root mean square error of approximation (RMSEA). CFI and AGFI larger than 0.90 and NNFI and GFI larger than 0.95 indicate a relatively good model fit. The *χ*^2^/*df* assessed the model's fit by comparing the obtained sample correlation matrix with the correlation matrix estimated under the model. Small *χ*^2^/*df* values indicate a good fit, reflecting the small discrepancy between the structure of the observed data and the hypothesized model. Other fit indices were considered because the *χ*^2^/*df* is extremely sensitive to sample size; considering model complexity, values smaller than 5 indicate an increasingly good fit. The RMSEA reflects how close the model fit approximates a reasonably fitted model, indicating a good model fit with values <0.05.

#### 2.9.4. Discriminative ability analysis

Two-sample *T*-test was carried out to compare scores on different dimensions based on an individual's sex, age group, one-child family or not, and residence. One-way ANOVA was conducted to compare individual scores on different dimensions based on caregivers' education, monthly per capita household income categories, and asthma control.

All statistical analysis was performed using SPSS 23.0 software. The normality of the data was evaluated using descriptive evidence from a one-sample Kolmogorov–Smirnov test. Quantitative and qualitative data are expressed as the “mean ± SD”, as well as frequencies and percentages. *P*-values <0.05 was considered to be significant.

## 3. Results

### 3.1. General characteristics of participants

The demographic characteristics of the three samples are shown in Table 1. 240 caregivers were enrolled using stratified sampling methods for the first investigation, in which 212 (88.3%) of their questionnaires were valid. A total of 503 caregivers were enrolled for the second round, with 428 (85.1%) valid questionnaires.360 caregivers were enrolled for the third round, with 326 (90.6%) valid questionnaires. The questionnaires were judged invalid if they were filled incorrectly or incompletely.

**Table 1:** Demographic characteristics of the children with asthma and their caregivers’ samples in three investigations.

Group	Sample 1 (*n* = 212)	Sample 2 (*n* = 428)	Sample 3	
			A (*n* = 326)	B (*n* = 60)
**Sex**				
Male	145 (68.40)	278 (64.95)	210 (64.42)	41 (68.33)
Female	67 (31.60)	150 (35.05)	116 (35.58)	19 (31.67)
**Age Group**				
2–4 years old	131 (61.79)	236 (55.14)	177 (54.29)	27 (45.00)
5–7 years old	81 (38.21)	192 (44.86)	149 (45.71)	33 (55.00)
**One child family**				
Yes	117 (55.19)	238 (55.61)	189 (57.98)	32 (53.33)
No	95 (44.81)	190 (44.39)	137 (40.02)	28 (46.67)
**Residence**				
Urban	124 (58.49)	291 (67.99)	219 (67.18)	45 (75.00)
Rural	88 (41.51)	137 (32.01)	107 (32.82)	15 (25.00)
**Father's Educational Background**				
Junior high school or less	40 (18.87)	80 (18.69)	60 (18.40)	7 (11.67)
Senior high school	61 (28.77)	106 (24.77)	81 (24.85)	21 (35.00)
College/university or more	111 (52.36)	242 (56.54)	185 (56.75)	32 (53.33)
**Mother's Educational Background**				
Junior high school or less	37 (17.45)	80 (18.69)	59 (18.10)	10 (16.67)
Senior high school	81 (38.21)	95 (22.20)	74 (22.70)	20 (33.33)
College/university or more	94 (44.34)	253 (59.11)	193 (59.20)	30 (50.00)
**Monthly per capita income** *				
Less than $750	137 (64.62)	188 (43.93)	147 (45.09)	22 (36.67)
$750–$1,500	64 (30.19)	177 (41.36)	132 (40.49)	28 (46.67)
More than $1,500	11 (5.19)	63 (14.72)	47 (14.42)	10 (16.67)
**Asthma Attack** (**the last 3 months**)				
Yes	113 (53.30)	206 (48.13)	155 (47.55)	24 (40.00)
No	99 (46.70)	222 (51.87)	171 (52.45)	36 (60.00)

Note: Data are present as *n* (%).

* $750 is approximately equal to 5,000 RMB, and $1,500 is approximately equal to 10,000 RMB.

### 3.2. Item selection

The data from Sample 1 were used to analyze and select the items. A total of 22 items were deleted according to the exclusion criteria, and a trial questionnaire containing 50 items was created. The data from Sample 2 were used to analyze and select the items. A total of 9 items were deleted according to the same criteria, and a final ACPRO questionnaire consisting of 6 dimensions and 41 items was formed.

### 3.3. Structure of the questionnaire

The data from the second investigation was selected for EFA. The Kaiser–Meyer–Olkin of the sample was 0.861 (greater than 0.6), the approximate chi-squared value for Bartlett's test of Sphericity was 3,149.08 (*P *< 0.05), and all results indicated that the data were fit for EFA. The parallel analysis plot showed that 5 factors should be extracted, and the cumulative variance contribution rate was 51.63%. However, some factors showed disparities. For ease of interpretation, according to the principle of eigenvalue greater than 1, we extracted 6 factors. The results of the EFA demonstrated that the variances of each factor explained were 25.86%, 9.38%, 6.84%, 5.22%, 4.33%, and 3.34%, and all the 6 factors explained 54.96% of the variance in the 34 items. The items' factor loadings were greater than 0.4 (Table 2).

**Table 2:** Items and factor loading of the final questionnaire reported by caregivers (*n* = 428).

Factors and items	Loading
**Factor 1** (**Eigenvalue = 8.79, Variance Contribution Rate = 25.86%**)	
My child's ability to take care of himself is the same as that of his age	0.647
My children can participate in the daily activities and exercises that other children can participate in	0.692
My child is able to keep balance when playing or doing some physical activities	0.751
My children will soon become familiar with the children they meet for the first time	0.736
My children can express their ideas and opinions clearly	0.801
When communicating with others, my child can respond in time	0.785
My child has one or several favorite children	0.761
**Factor 2** (**Eigenvalue = 3.19, Variance Contribution Rate = 9.38%**)	
My child will complain of discomfort or pain	0.553
My children feel different from other children	0.667
My children will be unhappy because they can't play with their friends	0.570
My child is reluctant to play with other children after illness	0.623
Because of asthma, my child is timid and seldom speaks in front of strangers	0.582
Because of asthma, my children will suffer from being unable to do what they like	0.731
My kids don't want to use inhalers or asthma drugs at school or outside	0.553
When he(she) went to the hospital for examination or treatment, my child showed negative emotions	0.409
**Factor 3** (**Eigenvalue = 2.33, Variance Contribution Rate = 6.84%**)	
Because of respiratory symptoms such as coughing, wheezing and suffocating, my child will complain about physical fatigue, tiredness, etc	0.527
My child will suddenly feel suffocated and out of breath	0.522
My child doesn't sleep well because of breathing problems (such as cough, chest tightness, shortness of breath, asthma attack, etc.)	0.672
A little more activity, my child coughs and sometimes can't breathe	0.607
My child will wake up in the morning with cough, chest distress, shortness of breath, asthma attack and other symptoms	0.731
My child will have cough, chest distress, shortness of breath, asthma attack and other symptoms at night	0.750
**Factor 4** (**Eigenvalue = 1.77, Variance Contribution Rate = 5.22%**)	
Some of our family's living habits have changed since our children got sick	0.680
I'm worried about my child's asthma attack	0.666
In order to take care of my children, I try to reduce social activities	0.740
The atmosphere in our family is not as lively as before after the child's illness	0.677
My work has been affected by my child's illness	0.775
**Factor 5** (**Eigenvalue = 1.47, Variance Contribution Rate = 4.33%**)	
I need to minimize my children's daily outdoor activities	0.719
My child can't do strenuous exercise	0.606
I will restrict my children from playing with other classmates or children	0.692
I'm afraid to take my children on a trip	0.635
**Factor 6** (**Eigenvalue = 1.14, Variance Contribution Rate = 3.34%**)	
In the environment of pungent smell, cigarettes or perfume, my child will suffer from cough, runny nose and sneezing.	0.672
When the weather suddenly gets cold, my child will have severe cough and respiratory symptoms	0.549
My child will suddenly have cough, stuffy nose, runny nose, sneeze, itchy nose or eyes	0.656
In a strange environment, my children are easily nervous or upset	0.506

The specific definition for each factor was established to understand the possible meaning of the items in each factor. Factor 1 contained 7 items and was named “Athletic and Communication Abilities (ACA)”, reflecting the asthmatic children's daily exercise, physical coordination, and communication ability with other children. Factor 2 contained 8 items named “Mentality and Emotion (ME)”, reflecting the asthmatic children's daily mood and the psychological and emotional changes caused by asthma. Factor 3 contained 6 items named “Asthma Symptoms (AS)”, describing the disease symptoms and physical changes caused by asthma in children. Factor 4 contained 5 items and was named “Family Influences (FI)”, reflecting the influences of asthma children's illness on family members and the family atmosphere. Factor 5 contained 4 items and was named “Activity Limitations (AL)”, reflecting the restrictions of caregivers on children's activities to reduce children's asthma attacks. Factor 6 contained 4 items and was named “Environmental Impacts (EI)”, reflecting the influences of environment and environmental change on children with asthma.

### 3.4. Reliability

All 326 participants in Sample 3 were included in an internal reliability analysis. The Cronbach's *α* coefficient for the total questionnaire was 0.91, and the 6 factors ranged from 0.70 to 0.88. The split-half reliability of the questionnaire was 0.75, and the 6 dimensions ranged from 0.68 to 0.83. The two-week test–retest reliability for the questionnaire (*n* = 60) was 0.74, with the 6 factors ranging from 0.68 to 0.82 (Table 3). These results showed that the questionnaire had good reliability.

**Table 3:** Reliability coefficients of the final asthmatic children patient reported outcomes questionnaire reported by caregivers in all dimensions (*n* = 326).

Dimensions	Cronbach's *α* coefficient	Split-half reliability coefficient	Test–retest reliability coefficient
Athletic and Communication Abilities	0.88	0.83	0.75
Mentality and Emotion	0.79	0.77	0.68
Asthma Symptoms	0.79	0.73	0.72
Family Influences	0.81	0.73	0.82
Activity Limitations	0.78	0.79	0.69
Environmental Impacts	0.70	0.68	0.78
Total	0.91	0.75	0.74

### 3.5. Validity

The content validity ratio was 0.6. The questionnaire items were easy to understand, and the mean time for completing the survey was 15.2 ± 3.4 min. The correlation coefficients ranged from 0.219 to 0.586 for dimensions of each other and 0.613–0.763 for dimensions between each dimension and the questionnaire. These results also suggested that dimensions were independent and representative of the questionnaire. The factor structure of the questionnaire was further evaluated by confirmatory factor analysis of Sample 3 (*n* = 326). The results (*χ*^2^/*df *=* *1.413 < 3, GFI = 0.894, AGFI = 0.870, NNFI = 0.943 > 0.9, CFI = 0.951 > 0.9, RMSEA = 0.036 < 0.05) met the statistical requirements, except for GFI and AGFI. Although GFI and AGFI did not align with the expected study results, they were close to the cutoff values, which was acceptable in the first compiled questionnaire. Also, previous studies have shown that RMSEA is less affected by the sample size but has more potential to affect the fitting index (26). All these results showed that the questionnaire had good construct validity.

### 3.6. Discriminative ability

The results of the discrimination validity analysis can be obtained from Table 4. The scores of Athletic and Communication Abilities and Family Influences were different for children of different genders (*P *< 0.05) and age groups (*P *< 0.05). There were significant differences in the Athletic and Communication Abilities, Environmental Impacts dimensions for children of different residences (*P *< 0.05) and monthly income per caregiver (*P *< 0.05). There were no significant differences in the scores of either dimension for children of one-child family or not (*P *> 0.05). The scores of each dimension (excluding Mentality and Emotion, Asthma Symptoms) were significantly different among children of different Father's Education (*P *< 0.05), and each dimension (excluding Mentality and Emotion, Asthma Symptoms, Environmental Impacts) were significantly different among children of different Mother's Education (*P *< 0.05). Moreover, the scores of all dimensions were significantly different for children with an asthma attack or not in the last three months (*P *< 0.05). The scores of all dimensions were significantly different among different asthma control states (*P *< 0.05), especially between well-controlled and uncontrolled.

**Table 4:** Comparison of each dimension score of patient reported outcomes questionnaire of children with asthma reported by caregivers among different characteristics of children and their caregivers in the third investigation of the questionnaire (*n* = 326).

Group	Athletic and Communication Abilities	Mentality and Emotion	Asthma Symptoms	Family Influences	Activity Limitations	Environmental Impacts
**Sex**						
Male	3.18 ± 0.75	3.27 ± 0.57	2.85 ± 0.64	2.19 ± 0.92	2.94 ± 0.83	2.23 ± 0.67
Female	3.35 ± 0.65^a^	3.20 ± 0.63	2.85 ± 0.68	1.89 ± 0.98^a^	2.92 ± 0.95	2.17 ± 0.77
**Age Group**						
2–4 years old	3.12 ± 0.78	3.24 ± 0.59	2.80 ± 0.62	2.00 ± 0.96	2.90 ± 0.87	2.18 ± 0.69
5–7 years old	3.39 ± 0.60^b^	3.26 ± 0.60	2.92 ± 0.68	2.18 ± 0.94^b^	2.97 ± 0.88	2.25 ± 0.74
**One Child Family**						
Yes	3.28 ± 0.68	3.24 ± 0.60	2.83 ± 0.65	2.06 ± 0.94	2.90 ± 0.90	2.16 ± 0.70
No	3.19 ± 0.76	3.26 ± 0.57	2.89 ± 0.66	2.12 ± 0.98	2.97 ± 0.84	2.28 ± 0.71
**Residence**						
Urban	3.33 ± 0.65	3.23 ± 0.59	2.80 ± 0.64	2.08 ± 0.96	2.97 ± 0.86	2.16 ± 0.70
Rural	3.07 ± 0.81^c^	3.28 ± 0.59	2.81 ± 0.67	2.10 ± 0.94	2.85 ± 0.89	2.33 ± 0.72^c^
**Father's Education**						
Junior high school or less	3.11 ± 0.82	3.35 ± 0.58	2.84 ± 0.69	2.09 ± 0.98	2.97 ± 0.78	2.42 ± 0.83^e^
Senior high school	3.11 ± 0.76	3.12 ± 0.64	2.76 ± 0.68	1.82 ± 0.95	2.54 ± 0.98^d^^,^^f^	2.17 ± 0.63
College/university or more	3.34 ± 0.65^d^^,^^e^	3.27 ± 0.57	2.90 ± 0.62	2.20 ± 0.93^e^	3.09 ± 0.80	2.16 ± 0.69
**Mother's Education**						
Junior high school or less	3.05 ± 0.82	3.31 ± 0.60	2.77 ± 0.67	1.95 ± 0.98	2.92 ± 0.78	2.39 ± 0.82
Senior high school	3.08 ± 0.76	3.14 ± 0.65	2.80 ± 0.70	1.91 ± 0.93	2.62 ± 1.05^g^^,^^i^	2.20 ± 0.67
College/university or more	3.37 ± 0.64^g^^,^^h^	3.27 ± 0.56	2.90 ± 0.63	2.19 ± 0.94^h^	3.05 ± 0.80	2.16 ± 0.68
**Monthly Income per Caregivers**						
Less than $750	3.10 ± 0.82	3.29 ± 0.58	2.84 ± 0.67	2.06 ± 0.97	2.88 ± 0.84	2.29 ± 0.67
$750–$1,500	3.32 ± 0.60^j^	3.24 ± 0.58	2.88 ± 0.63	2.16 ± 0.88	2.99 ± 0.87	2.24 ± 0.70
More than $1,500	3.46 ± 0.57^j^	3.14 ± 0.65	2.85 ± 0.67	1.94 ± 1.09	2.90 ± 1.00	1.89 ± 0.78^j^^,^^k^
**Asthma Attack** (**the last 3 months**)						
Yes	3.11 ± 0.57	3.15 ± 0.64	2.67 ± 0.67	1.98 ± 0.85	2.77 ± 0.88	1.96 ± 0.73
No	3.31 ± 0.61^l^	3.33 ± 0.53^l^	3.02 ± 0.59^l^	2.18 ± 0.92^l^	3.07 ± 0.84^l^	2.16 ± 0.69^l^
**Asthma Control**						
Well controlled	3.33 ± 0.67	3.34 ± 0.51	3.23 ± 0.49	2.19 ± 0.99	3.14 ± 0.84	2.42 ± 0.63
Partial controlled	3.29 ± 0.72	3.33 ± 0.52	2.92 ± 0.62^m^^,^^o^	2.14 ± 0.93	3.01 ± 0.80	2.26 ± 0.72
Uncontrolled	3.09 ± 0.74^m^	3.03 ± 0.70^m^^,^^n^	2.43 ± 0.58^m^^,^^n^	1.90 ± 0.95^m^	2.61 ± 0.93^m^^,^^n^	1.96 ± 0.70^m^^,^^n^

^a^ *P *< 0.05 vs. Male.

^b^ *P *< 0.05 vs. 2–4 years old.

^c^ *P *< 0.05 vs. Urban.

^d^ *P *< 0.05 vs. Junior high school or less (Father's Education).

^e^ *P *< 0.05 vs. Senior high school (Father's Education).

^f^ *P *< 0.05 vs. College/university or more (Father's Education).

^g^ *P *< 0.05 vs. Junior high school or less (Mother's Education).

^h^ *P *< 0.05 vs. Senior high school (Mother's Education).

^i^ *P *< 0.05 vs. College/university or more (Mother's Education).

^j^ *P *< 0.05 vs. Less than $750.

^k^ *P *< 0.05 vs. $750–$1,500.

^l^ *P *< 0.05 vs. Yes.

^m^ *P *< 0.05 vs. Well controlled.

^n^ *P *< 0.05 vs. Partial controlled.

^o^ *P *< 0.05 vs. Uncontrolled.

## 4. Discussion

The goal of this study was to describe the development, and preliminary evaluation of the Patient Reported Outcomes Questionnaire of Children with Asthma among Chinese children from 2 to 7 year-old, based on caregivers' reports. Preliminary evidence showed that the Questionnaire had good test–retest reliability and construct validity. As previous similar questionnaires have been mainly established for developed countries such as European and American countries, the 34 items and 6 dimensions of the Questionnaire allowed researchers to measure the patient-reported outcomes for Chinese children with asthma.

Based on the construction of the conceptual model of patient-reported outcomes reported by caregivers, this study completed the development and evaluation of the questionnaire by developing an item pool, experimental investigation, and three rounds of the formal investigation. We set up 8 pre dimensions and then maintained 6 dimensions for the formal questionnaire using parallel and exploratory factor analyses, with children's growth and asthma treatment removed. Although children's growth is affected by asthma to some extent, caregivers’ understanding of their children's growth is one-sided, and caregivers cannot accurately define their children's growth and development level when compared with other children of the same age. The aspect of asthma treatment mainly reflected children's response to medication and the frequency of treatment. Most of these contents were completed by professional doctors, not relating to children's overall quality of life or the impact of daily symptoms. Therefore, the formal questionnaire did not include these two dimensions in the factor analysis.

There are also some differences between the dimensions of the formal questionnaire and reference questionnaires. Compared with PAQLQ (9), it includes all dimensions in the questionnaire with three more dimensions: Family Influences, Environmental Impacts, and Athletic and Communication Abilities. Compared with CHSA-P (12), although there is no family mental health, it includes dimensions of Family Influences and Environmental Impacts. The questionnaire developed in this research mainly reflected the health status of children with asthma through the outcomes reported by their parents. In contrast, the parent's version of the CHSA mainly focused on children and family. Compared with the Pediatric Asthma Health Outcome Measure (PAHOM) (27), there are more dimensions of Athletic and Communication Abilities, Family Influences, and Environmental Impacts. These differences may be because most Chinese families only have one child. Hence, caregivers pay more attention to children's daily physical health, especially for children with diseases.

The study found differences in the dimensions of Athletic and Communication Abilities among children with asthma of different ages, residences, and monthly family incomes. The stronger the children's athletic ability, communication ability, and willingness, the higher their quality of life. These results are consistent with Banjari et al. (28), which suggested that children with older age, higher family income, and better living conditions would have better asthma control and higher quality of life. Moreover, gender dramatically influences the quality of life of children with asthma. This study showed that girls scored higher in the Athletic and Communication Abilities dimension, while boys scored higher in the Family Influences dimension. Children's physical fitness and asthma symptoms are greatly affected by the environment. Children with higher family incomes would have an excellent daily living environment and be more easily affected by the external environment, with lower scores for the environmental impacts dimension.

Caregivers' educational level significantly influenced Athletic and Communication Abilities, Family Influences, and Activity Limitations dimensions. Specifically, the higher the educational level of caregivers, the higher the children's scores in those dimensions, corresponding to the research results of Florinda et al. (29). Caregivers with higher educational levels may have a better educational style and a better understanding of asthma, and their children might receive treatment continuously to achieve a good therapeutic effect. The caregiver's educational level is also closely related to the family environment or influence. Families with high education levels also generally have a higher quality of life, which can provide a better living environment for children. In addition, the caregiver can communicate better with the doctor throughout the disease's treatment process and better understand the treatment. A good family environment provides ideal conditions for treating and recovering asthma in children, and the caregiver can take care of children with asthma more carefully and strictly follow the medical prescriptions for treatment. Results concerning children with or without asthma attacks in the previous three months demonstrated differences in all dimensions of the questionnaire, and the group of children with asthma attacks had higher scores in all dimensions than those without asthma attacks, which is consistent with the research results of Alfredo et al. (30), Moreover, the questionnaire has a good distinction in identifying asthma control levels.

The participants of this survey were patient's caregivers from large hospitals in Shaanxi province, which located in the middle of mainland China. There are more than 40 ethnicities (56 ethnicities of Chinese in total) settled in Shaanxi and its capital city, Xi'an is the biggest hub city in Northwestern region with outstanding medical resources to attract patients all around China. Hence, based on the diversity of patients' background ethnically and geographically, we could believe that the participants of this study can be a good representative of Chinese in general. Since the tool was developed based on children with asthma highlighted in different cultures and nationalities across China with vast territory, the utility of this tool can be expanded to children with asthma in other regions of China in the future.

We acknowledged that this study had some limitations. First, this questionnaire's reliability, validity, measurement invariance, and applicability in other geographical groups must be further confirmed. Second, cultural background, habits and customs have undoubtedly impact patient-reported outcomes of children with asthma, so it is essential to explore those measurement properties.

## 5. Conclusion

To our knowledge, this study is the first time a caregiver's proxy-reported measure of children with asthma was developed in China. The questionnaire includes 34 items and 6 dimensions. The 6 dimensions are Athletic and Communication Abilities, Mentality and Emotion, Asthma Symptoms, Family Influences, Activity Limitations, and Environmental Impacts. It provides a theory-based tool for assessing patient-reported outcomes of children with asthma, and preliminary evidence demonstrates good reliability, construct validity, and discriminative ability in samples of asthmatic children. Future studies should be conducted to examine and confirm the existing findings in different Chinese populations with larger sample sizes. Additional studies may also be needed to explore the possible applications of the questionnaire in guiding and evaluating the future treatment and control levels of children's asthma in China.

## Data Availability

The raw data supporting the conclusions of this article will be made available by the authors, without undue reservation.
